# Wheat germ, a byproduct of the wheat milling industry, as a beneficial source of anti‐aging polyamines: A quantitative comparison of various forms

**DOI:** 10.1002/fsn3.3650

**Published:** 2023-09-07

**Authors:** Maryam Mohajeri, Seyed Abdulmajid Ayatollahi, Farzad Kobarfard, Mohammad Goli, Maryam Khandan, Shaya Mokhtari, Mahmoud Khodadoost

**Affiliations:** ^1^ Phytochemistry Research Center Shahid Beheshti University of Medical Sciences Tehran Iran; ^2^ Department of Pharmacognosy, School of Pharmacy Shahid Beheshti University of Medical Sciences Tehran Iran; ^3^ Department of Medicinal Chemistry, School of Pharmacy Shahid Beheshti University of Medical Sciences Tehran Iran; ^4^ Department of Food Science and Technology, Laser and Biophotonics in Biotechnologies Research Center, Isfahan (Khorasgan) Branch Islamic Azad University Isfahan Iran; ^5^ Central Research Laboratories Shahid Beheshti University of Medical Sciences Tehran Iran; ^6^ Department of Traditional Medicine, School of Traditional Medicine Shahid Beheshti University of Medical Sciences Tehran Iran

**Keywords:** HPLC‐MS/MS method, isobutyl chloroformate, polyamine extraction, wheat germ

## Abstract

Polyamines have received a lot of attention since the 1990s because of their anti‐aging, anti‐chronic disease, and proliferative effects. Wheat germ was reported as one of the natural sources of high polyamine, especially spermidine. The current study used three types of wheat germ: group A was industrially separated germ from whole grain, group B was the commercially available germinated wheat germ, and group C was manually separated wheat germ from germinated grain. The polyamine content of putrescine, spermidine, and spermine has been determined using a simplified isocratic LC–MS/MS method. An optimized extraction procedure was performed on all seven samples for obtaining a polyamine‐enriched extract. The three dominant carbomylated polyamines were identified by analyzing the extracted samples in order to determine their relative abundance. Wheat germ powders contain the highest amount of polyamines (220–337 μg/g) of which spermidine is one of the most important. Germinated wheat grains, on the other hand, contain the least amount of this polyamine. The commercially available separated wheat germs are suggested as a good nutrition source of these polyamines.

## Graphical abstract

Comparison of efficiency of four different polyamine extraction methods. Highest yield extraction of wheat germ (WG) free polyamines under mild condition. Optimization of polyamine derivatization by isobutyl chloroformate. First report on WG polyamine determination by a simple isocratic LC–MS/MS method. WG as a good nutritional source for improving cell proliferation and anti‐aging.

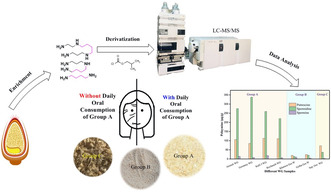

## 1 INTRODUCTION

Aging is a progressive, harmful process caused by various factors, affecting various systems like sensation, cognition, memory, and motor control. It leads to loss of functions in these systems. Cellular metabolism generates oxidative products, causing oxidative stress, DNA damage, and apoptosis. This process affects all organism levels and can have severe consequences (Alvarado et al., 2011; Bianchi et al., 2007; Bosch, 2016; Latash et al., 2010; Okita et al., 2007; Saeki et al., 2011; Singh et al., 2011). Polyamines are stable, protonated molecules that bind to DNA and RNA macromolecules at physiological pH. They suppress DNA and RNA damage, potentially contributing to anti‐aging effects. Polyamines influence aging through various mechanisms due to their interactions with various molecules. Aging reduces polyamine synthesis, leading to mutagenesis and cell death (Agostinelli et al., 2010; Handa et al., 2018; Madeo et al., 2018; Minois, 2014). Polyamine‐rich foods are beneficial for aging retardation and delay, as long‐term oral consumption leads to high levels of polyamines in the blood, increasing cell proliferation, longevity, skin rejuvenation, and hair and nail growth (Oryza Oil & Fat Chemical Co, 2011). This also lowers the risk of cardiovascular disease, chronic neurological diseases, chronic inflammatory diseases, rheumatoid arthritis, inflammatory bowel disease, osteoporosis, and cancer (Galasso et al., 2023; Karouzakis et al., 2012; Makletsova et al., 2022; Novita Sari et al., 2021; Polis & David Karasik, 2021; Saiki et al., 2019; Tong & Hill, 2017). Spermidine, spermine, and putrescine are the most abundant polyamines in living organisms and can be found in various foods (Nonami, 2002). Overall, polyamine‐rich foods are essential for maintaining overall health and well‐being. Studies on determination of polyamine levels in various foods have demonstrated that wheat germ is a promising source. Polyamines in plants vary by species, with putrescine found in fruits and spermidine in grains. Wheat germ contains the highest amount of polyamines, particularly spermidine, among cereals (Moret et al., 2005; Muñoz‐Esparza et al., 2019; Nishibori et al., 2007; Nishimura et al., 2006; Tong & Hill, 2017; Table 1). Wheat germ, a small part of wheat, only 2.5% of the total seed weight, contains high polyamine content and is responsible for storing genetic information for growth. It contains proteins, vitamins, essential fatty acids, and polyamines. Wheat germ is sold in two forms: germinated wheat powder with gluten and starch and separated wheat germ without other grain parts (Pringle et al., 2005). No comparative study has been published on the polyamine content of different parts of wheat. Polyamine analysis in various tissues has been extensively studied, with various extraction protocols for polyamine extraction. These extraction methods performed with perchloric acid, trichloroacetic acid, and citric acid at a mild pH (Dai et al., 2014; Kitazawa & Yanagidani, 2014; Magnes et al., 2014; Pringle et al., 2005). Numerous articles have been published on polyamine analysis in various tissues, and polyamines have been isolated and quantified using techniques like TLC, HPLC, GC–MS, LC–MS/MS, and capillary electrophoresis (CE; Cunha et al., 2011; Dai et al., 2014; Flores & Galston, 1982; Hammond & Herbst, 1968; Herbst, 1968; Kaneta, 2018; Kmieciak et al., 2023; Lee et al., 2021; Lu et al., 2022; Magnes et al., 2014; Pringle et al., 2005). Polyamine amounts in plant and animal tissues and biological fluids can be determined using two methods: derivatization‐free methods using strong acids like heptafluorobutyric acid or trifluoroacetic acid and monitoring by MS/MS system (Gosetti et al., 2013; Häkkinen et al., 2008; Ochi, 2019; Sánchez‐López et al., 2009), and analytical methods with derivatization using isobutyl chloroformate (IBCF), dansyl chloride, or benzoyl chloride using UV, fluorescence, or MS/MS detectors (Cunha et al., 2011; Esatbeyoglu et al., 2016; Jain et al., 2013; Lee et al., 2021; Pringle et al., 2005; Samarra et al., 2019). Each method has advantages and disadvantages, and the appropriate analysis method should be chosen based on the sample type, matrix effect, and reproducibility (Dai et al., 2014; Lee et al., 2021; Magnes et al., 2014; Muñoz‐Esparza et al., 2019). The determination of polyamines is affected by interference effects and their high polarity, necessitating strong acids to retain them (Lee et al., 2021). Lee et al. (2021) developed a simultaneous analysis method for serum polyamines and steroids using LC–MS/MS and IBCF as derivatization agents, favored for its quick and simple process (Lee et al., 2021). The study aimed to determine free polyamines in wheat germ forms A: industrially separated germ from whole grain, B: commercially available germinated wheat germ, and C: manually separated wheat germ from germinated grain. The simple and sensitive LC–MS/MS analysis method was developed, suggesting that the polyamine‐rich extract from wheat germ could be a nutraceutical with high polyamine content.

**TABLE 1:** Comparison of polyamine levels in various plant foods.

Extraction from	Putrescine (μg/g)	Spermidine (μg/g)	Spermine (μg/g)	Ref.
Fruits Apple, avocado, banana, cherry, kiwi, mandarin, orange, pear, peach, pineapple, strawberry, fruit juices	nd − 136.98	1.009–14.33	nd − 5.06	Flores and Galston (1982), Nishimura et al. (2006)
Vegetables Broccoli, cabbage, cauliflower, carrot, celeriac, courgette, cucumber, eggplant, green beans, green pepper, lettuce, mushroom, onion, potato, spinach, tomato.	0.5–69.99	1.008–58.18	nd − 10.93	Flores and Galston (1982), Nishimura et al. (2006), Nishibori et al. (2007), Moret et al. (2005)
Legumes and soybean products Chickpeas, lentils, peas, white beans, red kidney beans, soybean, soybean sprouts, soybean milk, tofu, soy sauce, miso	nd − 46.28	0.146–208.33 (Zhao et al., 2017)	nd − 68.99	Flores and Galston (1982), Nishibori et al. (2007), Nishimura et al. (2006)
Nuts and oilseeds Almonds, chestnuts, pistachios, seeds	2.99–43	5.99–55.99	12.75–33.39	Flores and Galston (1982), Nishimura et al. (2006)
Cereals Rice, wheat germ	0.2–62	0.409–356.3	nd − 146.09	Flores and Galston (1982), Moret et al. (2005), Nishibori et al. (2007)

## 2 MATERIALS AND METHODS

### 2.1 Chemicals

Sigma Aldrich provided spermidine standard (99% purity), putrescine standard (99% purity), spermine standard (99% purity), 1, 6‐diaminohexan (internal standard), and IBCF as derivatization agents. Merck provided the ethanol and acetonitrile, both of which were HPLC grade. The analytical grade chemical reagents, disodium hydrogen phosphate (Merck), and sodium hydroxide (Merck) were used without further purification.

### 2.2 Plant material

Sample preparation was conducted on three various kinds of wheat germs including (Table 2):

1. Group A) Wheat germ separated from the grain in milling process.
2. Group B) The germinated wheat grains.
3. Group C) Wheat germ separated manually from the germinated wheat grain (G‐C).

**TABLE 2:** List of various kinds of wheat germs analyzed in the present study.

Group	Sample type	Sample code	Brand name	Country	City
A	Wheat germ powder	GA‐G	Good Mills	Germany	Hamburg
		GA‐A	Atrineh	Iran	Isfahan
		GA‐KJ	Kalil Javaneh	Iran	Tehran
		GA‐R	Reyhaneh	Iran	Tehran
B	The germinated wheat grains	GB‐G	Golha		Hamburg
		GB‐Kh	Khatoon	Iran	Isfahan
C	Wheat germ separated manually from the germinated wheat grain	GC	–	–	–

### 2.3 Sample preparation

#### 2.3.1 Extraction of polyamines

A 25°C water bath was used to stir 1 g of wheat germ powder with 5 mL of a 2:3 ethanol/water solution for an hour. The mixture was centrifuged at 177,408 g for 20 min, and the supernatant was discarded. The plant residue was mixed with 5 mL of water and stirred for an hour at 25°C. The solution was adjusted to a pH of 4.0, and the resulting solution was mixed with more citric acid. After centrifuging at 177,408 g for 20 min, the supernatant was separated and evaporated on a rotary evaporator to obtain a dry powder (Mohajeri et al., 2023).

#### 2.3.2 Derivatization of standard and unknown samples

One millilitter sample solution was transferred to a 15 mL Falcon tube containing 2 mL of toluene and 10 μL of 1, 6‐diaminohexane as an internal standard. The solution was mixed with 0.5 M phosphate buffer and 100 μL of IBCF. After vortex‐mixing of the Falcon flask for 10 min, disodium sulfate anhydrous (Na_2_SO_4_; 2 g) was added to the solution, mixed, and centrifuged (10 min at 15,092 g). One millilitter of the organic layer and 1 mL of alkaline methanol (containing 2 g/mL potassium hydroxide) were added to another Falcon flask. Three milliliters of a 5 M sodium hydroxide (NaOH) solution were added and the tube had been vortex‐mixed for 5 min. The mixture was centrifuged, and 100 μL of the organic layer was transferred to a vial and evaporated to dryness under a nitrogen gas stream. The dried residue was dissolved in 1 mL of acetonitrile and injected into LC–MS/MS (Cunha et al., 2011).

### 2.4 LC–MS/MS analysis

The LC–MS/MS analysis of samples was performed using a HPLC separation module (Agilent series, 1200, Germany) equipped with a quaternary solvent delivery system, degasser, auto sampler, and column heater coupled with a Triple Quadrupole LC–MS (Agilent Technologies), and separation was carried out using a MZ Analysis C18 column (150 mm, 4.6 mm, particle size: 5 m). The mobile phase for elution was established in an isocratic mode, water with 0.1% formic acid (as phase A) and acetonitrile with 0.1% formic acid (as phase B) and eluted with 18.5% A/81.5% B at a flow rate of 0.35 mL/min. Each sample was injected in 5 μL. Temperatures of 35 and 25°C, respectively, were maintained for the column and auto sampler. The product ion spectra were used to choose the proper precursor and product ions. The most abundant product ion was selected as quantitation ion in multiple reactions monitoring (MRM) mode analysis. Mass spectrometry parameters included a spray voltage of 5.0 kV, capillary temperature of 320°C, gas flow rate of 8 L/min, and capillary voltage of 32 V. Positive ion MRM detection was used to find all analytes, and the analytical conditions were optimized for each analyte (Table 3; Mohajeri et al., 2023).

**TABLE 3:** MRM acquisition settings of the IBCF polyamines and internal standard.

Compound name	Number of IBCF groups	MW	MRM transition (*m*/*z*)	Normalized CE	Fragmentor voltage	Retention time (min)
Putrescine	2	288.2	288.2 > 115.1	10	20	6.5
			288.2 > 215.1	5	20	6.5
Spermidine	3	445.2	446.2 > 372	10	100	8.5
			446.2 > 298	10	100	8.5
Spermine	4	602.66	603.7 > 529.4	10	100	13
			603.7 > 455.3	20	100	13
1,6‐diaminohexane	2	316.2	317.2 > 243.1	5	100	7.5
			317.2 > 143.1	15	100	7.5

#### 2.4.1 Calibration curve

A calibration curve was created by preparing standard polyamine solutions. A 1 mg/mL polyamine stock solution was prepared, and Working Standard solutions at the concentration levels of 0.2, 0.4, 0.6, 0.8, and 1 mg/mL for spermidine and 0.05, 0.1, 0.2, 0.4, 0.6, 0.8 and 1 mg/mL for spermine and putrescine were prepared. 10 μL of 1, 6‐diaminohexan (internal standard) solution was added to 1 mL of each standard sample. Unknown samples were diluted with water (1:10) prior to the injection to minimize the ion suppression. The calibration curves were constructed by plotting peak area ratios (compound peak area/internal standard peak area) against the standard concentrations.

#### 2.4.2 Method validation

This approach was validated using linearity test, precision and accuracy of inter‐day and intra‐day runs, and limit of quantification (LOQ). The correlation coefficient (*R* ^2^) was used to assess linearity. The LOQ was the lowest quantifiable concentration with a signal‐to‐noise ratio of more than 10. Utilizing quality control (QC) samples of each polyamine at 3–5 various concentrations, accuracy and precision were assessed. Precision was measured as the relative standard deviation (% RSD), while accuracy was computed as the relative error (% bias). While inter‐day validation required sample analysis on three different days, intra‐day validation was confirmed by three examinations of samples on the same day. The matrix effect was assessed by adding a standard solution (100 μL of standards to 0.1 g of enriched powder and distilled water to 1 mL) to the samples using the following equation, the matrix effect was assessed:

(1) $$\text{Matrix effect}\;\left(\%\right)=\left[\frac{\left(A-B\right)}{B}\right]\times 100$$

where *A* and *B* are as follows:

(2) $$A=\frac{\text{Spiked solution peak area}}{\text{IS peak area}}$$

(3) $$B=\frac{\text{Standard solution peak area}}{\text{IS peak area}}$$

### 2.5 Statistical analysis

Statistical SPSS software version 16 was used to conduct all tests in a completely randomized design (Chicago, SPSS Inc.). Weight of polyamine to weight of dried wheat germ powder was a determining factor. The current study was successful since it included seven treatments and three replications. At the 95% confidential level, Duncan's multiple range test revealed significant differences (*p* < .05).

## 3 RESULTS AND DISCUSSION

### 3.1 Extraction procedures

**FIGURE 1:**
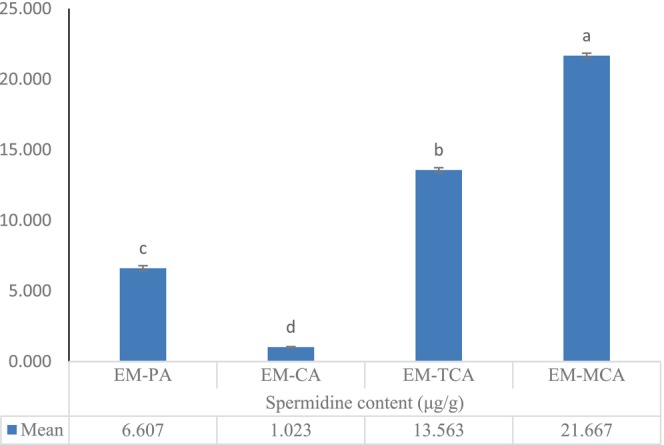
Selection of suitable polyamine extraction method of wheat germ. Data represents mean standard deviation for three replicates of the same sample (*n* = 3). Different superscripts in the same column indicate statistically significant differences (*p* < .05).

The best procedure for completely extracting all polyamines from wheat germ was determined by testing various extraction techniques on the same samples up until there were none left in the plant tissue. Table 4 was used to categorize the polyamine extraction techniques into four groups: EM‐PA, EM‐CA, EM‐TCA, and EM‐CAM. The use of an acidic solution, such as perchloric acid, tricholoroacetic acid, or citric acid, forms the basis of all polyamine extraction techniques. The measurement of polyamines in plant tissues (Sánchez‐López et al., 2009), foods (Wu et al., 2018), human blood, and urine (Häkkinen et al., 2013; Lee et al., 2021; Magnes et al., 2014) has been the subject of numerous papers. Perchloric acid was recommended in these papers as the ideal solvent for polyamine extraction in its measuring techniques. Percholoric acid could not be used to prepare polyamine‐enriched extracts for oral formulations due to its toxicity and potent acidic properties. Therefore, using citric acid, an approved natural edible acid that can be consumed, under controlled conditions, we optimized the polyamine extraction. Another benefit of our suggested extraction process is the ability to remove additional biocompounds utilizing ethanol as an edible solvent (Kitazawa & Yanagidani, 2014). The optimal extraction technique was determined to be route EM‐CAM based on the extracted spermidine values shown in Figure 1. In the end, we suggest an ideal polyamine extraction method that is excellent for nutritious formulations due to its high efficiency and high net weight of polyamines in the extract.

**TABLE 4:** Reported extraction methods in order to determine the best enrichment method.

Method	Extraction method	Problems of method	Advantages of method	Ref
EM‐PA	Polyamine extraction by Percholoric acid 5%	Toxicity of perchloric acid Low polyamine weight ratio The need for neutralization and the high weight of the final polyamine enriched extract	Short reaction time	Dai et al. (2014), Magnes et al. (2014)
EM‐CA	Polyamine extraction by Citric acid 10%, 50%, 80%	The high weight of the polyamine enriched extract Low polyamine weight ratio	Use of an edible acid	
EM‐TCA	Polyamine extraction by Tricholoroactic acid 5%	Use of inedible acid and solvents The high weight of the polyamine enriched extract	Better efficiency comparing methods A and B Having less weight of dried extract than the previous methods	Regional (1972)
EM‐CAM	Polyamine extraction under mildly acidic conditions by citric acid with several clean up steps in laboratory scale	Long extraction time This method is not suitable for high weights of wheat germ powder in industrial scale	The low weight of the extract residue The highest polyamine weight ratio This method was the most appropriate extraction method for oral consumption	Kitazawa and Yanagidani (2014)

### 3.2 Derivatization and determination

**FIGURE 2:**
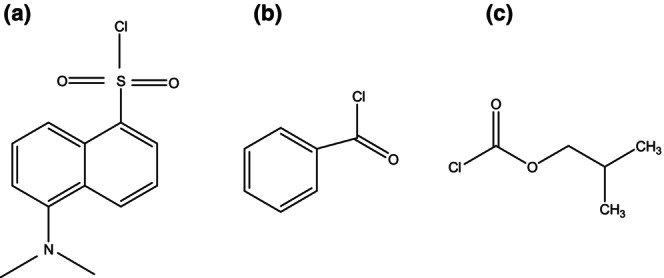
Structure of (a) Dansyl Chloride, (b) Benzoyl Chloride, and (c) Isobutyl Chloroform Derivatives.

There are typically two methods used to analyze extracted polyamines: derivatization free method and analysis method with derivatization. Table 5 provides a list of these strategies' advantages and disadvantages. Derivatization has benefits; hence, this strategy was chosen as the recommended option for the current investigation. Polyamines are easily eluted from reverse phase chromatographic columns because of their polar character. On the other hand, their low molecular weight will cause numerous interfering peaks to appear. It has been reported that pre‐ or post‐column derivatization using dansyl chloride, benzoyl chloride, ortho‐phthalaldehyde, IBCF, etc., can be used to quantify polyamines using HPLC equipped with a UV, fluorescence, or mass spectrometry detector (Kushad & Yelenosky, 1987; Lee et al., 2021; Sethi et al., 2011; Slocum et al., 1989; Widner et al., 2021). Figure 2 presents the chemical structures of the most popular derivatization reagents for reactions with polyamines. Benzoyl chloride derivatizes polyamines quickly (in about 10 min), but the low sensitivity of the UV detector to this molecule renders it undesirable. In addition, a gradient program with a lengthy run time must be used to perform the separation. Derivatization of dansyl chloride requires a gradient approach and a longer run time, and it takes more time (overnight at RT or 60°C). Therefore, because derivatization with IBCF requires less time for reaction and is compatible with the isocratic mobile phase system, derivatization with IBCF was chosen as the preferred approach. IBCF favors faster reactions than aromatic derivatization reagents like dansyl chloride and benzoyl chloride because of its aliphatic nature. As a result of their conjugation with an aromatic ring, the sulfonyl group in dansyl chloride and the carbonyl group in benzoyl chloride are both less electrophilic and less reactive when reacting with polyamine nucleophiles. Therefore, aliphatic reagents like IBCF make it easier to carry out nucleophilic substitution reactions.

**TABLE 5:** comparison of non‐derivative and derivative methods for analysis of polyamines.

Non‐derivative methods		Derivatization methods	
Advantages	Disadvantages	Advantages	Disadvantages
Simple one step process Higher sensitivity of the method	Just one detection method by MS Reduced performance of the column, reduced accuracy, and reproducibility due to strong acid utilization	Different detection methods (UV or Fluorescent detector or LC–MS/MS) Higher accuracy and reproducibility	Possible sensitivity reduction due to one extra step

**FIGURE 3:**
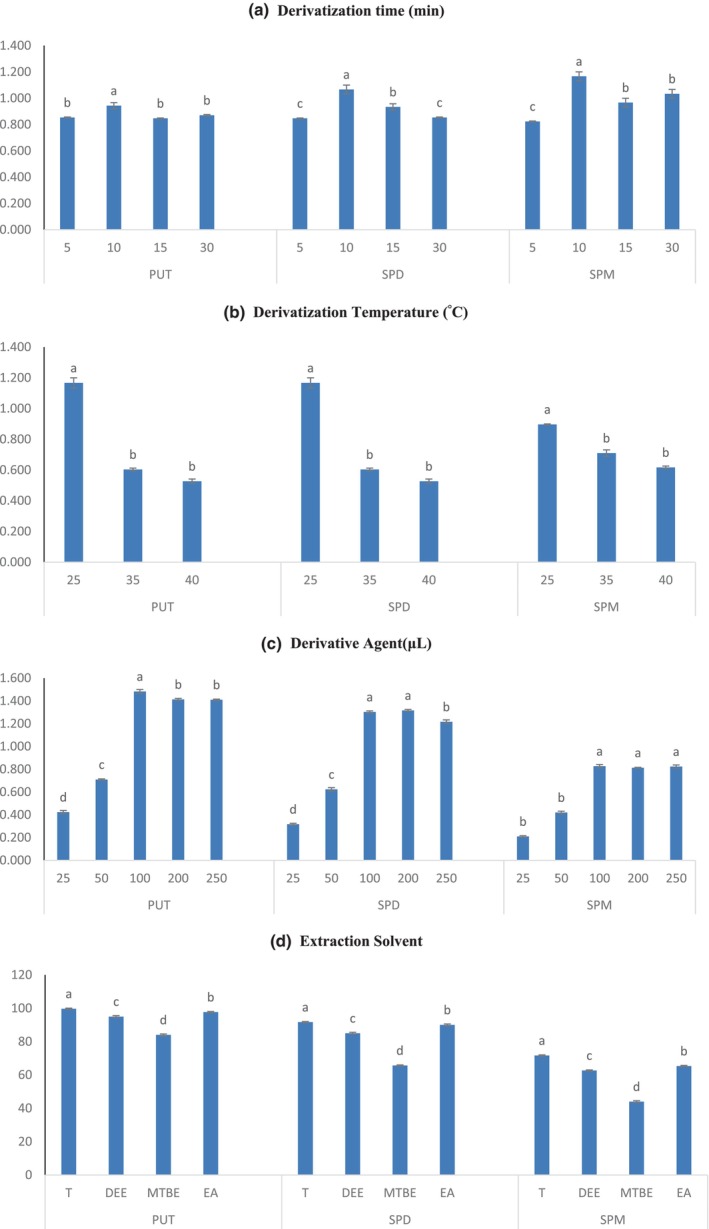
Comparison of the optimization procedures. Isobutyl chloroformate derivatization under varying conditions including reaction time (a), reaction temperatures (b), derivative agent (c), and extraction of liquid–liquid extraction solvents (d). Data represents mean ± standard deviation the ratio of polyamine weight to plant powder weight (a, b, c) and extraction yield (d) for three replicates of the same sample (*n* = 3). Different superscripts in the same column indicate statistically significant differences (*p* < .05) for optimized procedure.

We assessed and optimized the IBCF and polyamine's reaction conditions. On the polyamine‐derivatization reaction, the impacts of IBCF concentration, reaction time, temperature, and extraction solvent as factors were assessed. Different reaction times (5, 10, 15, and 30 min), reaction temperatures (25, 35, and 40°C), and quantities of derivatization solutions (25, 50, 100, 200, and 250 μL) were evaluated to find the reaction parameters that would produce the maximum derivatization efficiency. To improve the extraction efficiency of derivatized polyamine, four different extraction solvents were used: diethyl ether, ethyl acetate, methyl tert‐butyl ether, and toluene (T). The best extraction solvent was discovered to be toluene. The final setting called for 100 μL of IBCF, a reaction time of 10 min at 25°C, and toluene extraction. The significance of the parameters for each standard is shown in Figure 3.

### 3.3 Liquid Chromatography–Tandem mass spectrometry

**FIGURE 4:**
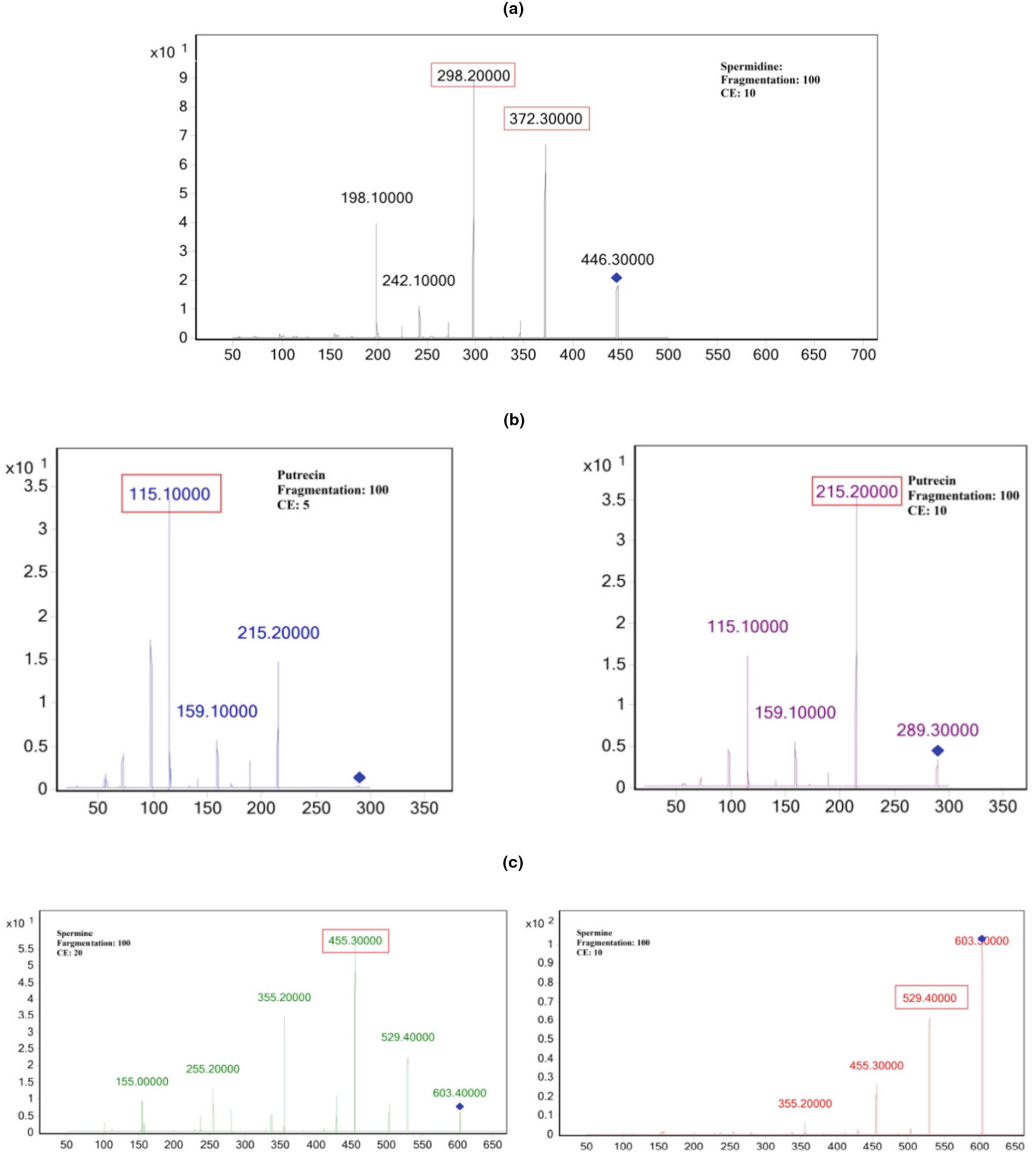
Positive‐ion MS/MS product ion mass spectra for (a) Spermidine (b) Putrescine (c) Spermine.

**FIGURE 5:**
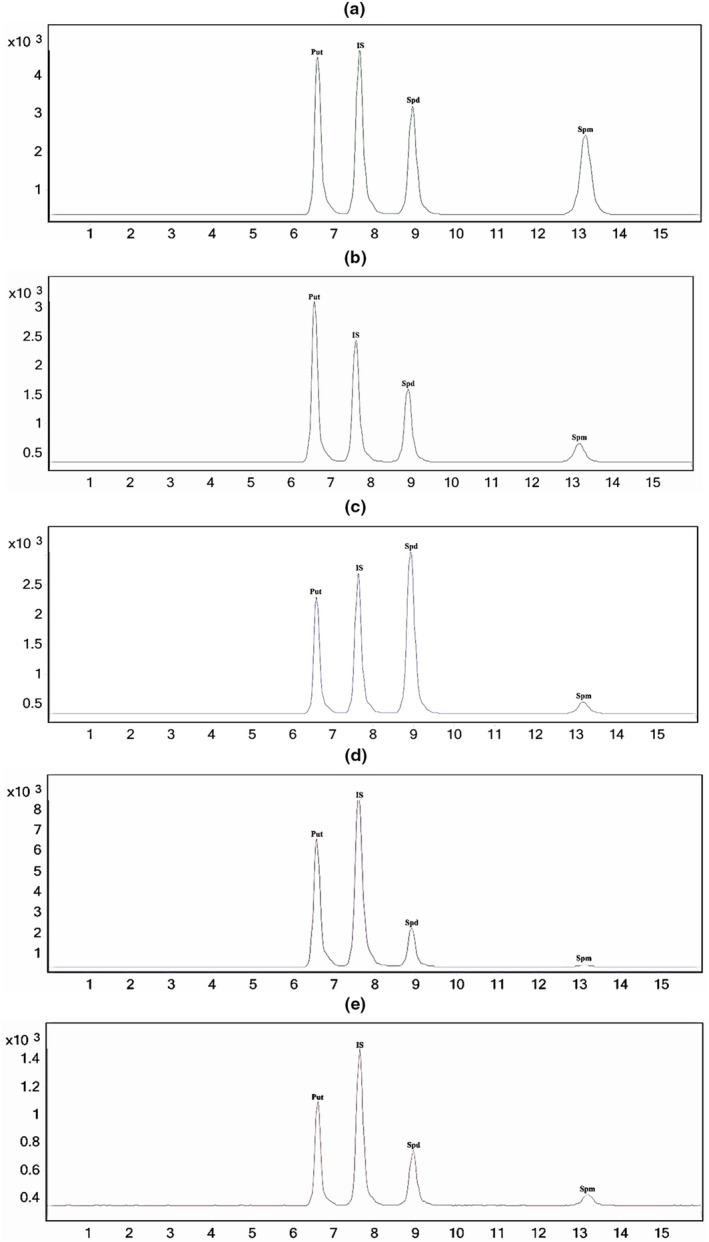
Chromatographic separation and identification of carbamylated polyamines. (a) Standards plus internal standard. Identification of polyamines (Putr, Spd, and Spm) in (b) Atrineh Wheat germ, (c) Germany Wheat germ, (d) Wheat germ, separated from germinated wheat, (e) Kalil Javaneh Wheat germ.

In order to identify polyamine isolated from wheat germs, the current study proposes a straightforward isocratic approach based on derivatization with IBCF on a reverse phase chromatographic column (C18) utilizing an Agilent HP series 1100 binary pump LC–MS/MS system. The residue is reconstituted in acetonitrile and injected onto the LC–MS after drying the derivatized sample. The autosampler was set to a temperature of 25°C, and column temperatures were adjusted at 35°C. The injection had a 5 μL volume. To obtain suitable retention times and resolution, acetonitrile and water that contained 0.1% formic acid were used as the mobile phases. With a flow rate of 0.35 mL/min for 16 min, the isocratic elution was performed with a fixed B concentration of 81.5%. By using ESI in the positive ion mode and MRM detection, the derivatized polyamines were separated. The [M + H] ^+^ ions were regarded as precursor ions in the MRM study. The [M + H ‐ OCH_2_C_3_H_7_]^+^ ion appeared as the basis peak for quantification in all derivatized polyamines. Based on the amount of amine groups in the compound's chemical structure and the product ion spectrum of polyamines shown in Figure 4, the primary product ions' *m*/*z* values were established. The most intense peaks in the product ion spectra of carbamylated polyamines are ion fragments of 298 and 372 (*m*/*z*) for spermidine, 115 and 215 (*m*/*z*) for putrescine, and 455 and 529 (*m*/*z*) for spermine. 1, 6‐diaminohexane was employed as the internal standard (IS) to measure polyamines and compensate for the matrix effect. We devised the analysis of polyamines utilizing an isocratic technique, a straightforward, practical, and quicker method than Lee et al works (Lee et al., 2021). The retention time for each polyamine rose as the number of amine groups increased, as seen in Figure 5.

All of the polyamines were detected in the positive ESI mode and by MRM technique. Direct injection of the standard solutions into the mass spectrometer using a syringe pump at a flow rate of 0.35 mL/min was used to optimize the precursor ions, product ions, fragmentor voltage (Fr), and collision energy (CE).

The standard solution's precursor ions were fragmented in order to optimize the MS parameters. Two ion transitions (product ions) with greater molecular masses and better intensities were chosen in order to reduce interferences. Each compound has a unique series of CE values and fragmentor voltages. Putrescine's lower molecular weight than spermine and spermidine allowed for the required ion transitions to occur at lower fragmentor voltages. Food residue analysis faces significant challenges due to matrix effects (Nasiri et al., 2021). Remediating the matrix effect was a key concern in this study due to the complexity of the wheat matrix. Polyamine standard solution was added to the samples in order to assess any potential matrix effects. In addition, 1, 6‐diaminohexane (internal standard) was added to the standard solutions and wheat germ samples to reduce measurement error caused by the matrix effect.

### 3.4 Method validation

To confirm the linearity of calibration curves, ratios of analyte peak area to internal standard peak area in the range of 0.05–1 mg/mL were plotted versus polyamine concentration. In three different days, three or four independent replicates of each of the seven concentration levels were performed. For all analytes, the regression equations were determined to be linear with an appropriate correlation coefficient, *R* ^2^ > 0.99. Table 6 displays the results. The developed method was validated by evaluating the accuracy and precision of QC samples in 3–5 concentrations, as shown in Table 6. The relative standard deviation (% RSD) was used to assess precision, while the relative error rate (% bias) was used to assess accuracy. The validity of the LC–MS/MS method was established because the accuracy and precision values were less than 20%. The LOQ for spermidine was set at 0.2 mg/mL, putrescine at 0.05 mg/mL, and spermine at 0.1 mg/mL (Table 7).

**TABLE 6:** Calibration range, linear regression equation, limits of quantification (LOQ), matrix effect, and recovery of polyamines.

Analytes	Calibration rang (mg/mL)	Linear regression equation	R^2^	LOQ (mg/mL)	Matrix effect (%)
putrescine	0.1–1	*Y* = 1.6433*x* + 0.2874	0.9957	0.1	19.06
spermidine	0.2–1	*Y* = 1.3317*x* + 0.3855	0.9982	0.2	15.36
spermine	0.05–1	*Y* = 3.0004*x* + 0.0714	0.9979	0.05	10.45

**TABLE 7:** Intra‐day and inter‐day validation of polyamines.

Analytes	QC concentration (mg/mL)	Intra‐day (*n* = 3)		Inter‐day (*n* = 3)	
		Accuracy (%bias)	Precision (%RSD)	Accuracy (%bias)	Precision (%RSD)
PUT	0.05	97.58	2.425	91.63	8.37
	0.2	91.73	7.04	92.86	7.135
	0.4	92.96	8.27	89.47	10.53
SPD	0.2	97.3	3.5	109.2	9.5
	0.4	105.8	1.5	112.4	4.47
	0.6	95.5	4.5	103.2	7.3
	0.8	98.9	1.12	103.7	4.8
SPM	0.1	108.2	8.23	113.13	13.15
	0.2	111.7	11.69	116.2	16.23
	0.4	104.3	4.32	104.06	3.79
	0.6	93.9	6.1	99.49	2.64
	0.8	88.6	11.5	95.9	4.14
	1	80.5	19.5	85.9	14.09

Matrix effects ranging from 10% to 19% were computed for all three analytes (Table 6). The relative standard deviation (% RSD) was used to assess precision, while the relative error rate (% bias) was used to assess accuracy. The intra‐day (*n* = 3) precision (% RSD) and accuracy (% bias) for spermidine were 1.5%–4.5% and 95.5%–105.5%, respectively, for spermine, 4.32%–19.5% and 80.5%–111.7%, and 2.425%–8.27% and 91.73%–97.58% for putrescine. Whereas the inter‐day (*n* = 3) precision and accuracy for spermidine were 4.47%–9.5% and 103.2%–112.4%, 2.64%–16.23% and 85.9%–116.2% for spermine, and 7.135%–10.53% and 89.47%–97.86% for putrescine (Table 7).

### 3.5 Polyamine concentrations in wheat germ samples

**FIGURE 6:**
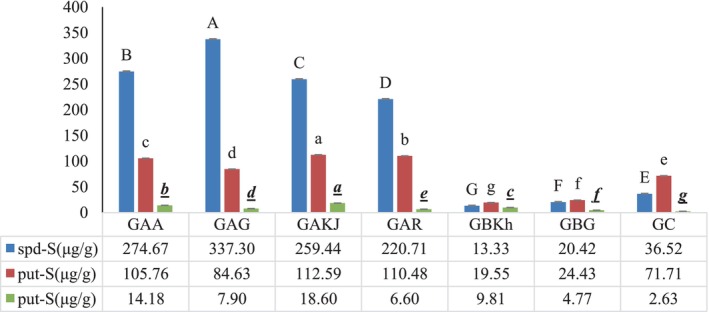
Comparison of the polyamine content in three groups of dried wheat germ powder. Data represents mean ± standard deviation for three replicates of the same sample (*n* = 3). Different superscripts in the same column indicate statistically significant differences (*p* < .05).

Polyamine levels in plant tissues have been measured in a variety of ways. According to published findings (Table 1), the largest concentrations of polyamines were found in fruits, legumes, and soybean products, and grains. Fruits had the largest concentration of putrescine, while grains had the highest concentration of spermidine (Lee et al., 2021; Magnes et al., 2014; Muñoz‐Esparza et al., 2019; Nonami, 2002; Yoshinaga et al., 1993). In the case of continuous oral polyamine consumption, they serve an important function in maintaining cell proliferation and prolonging lifespan (Oryza Oil & Fat Chemical Co, 2011). Thus, the polyamine concentration of several kinds of wheat germ, namely group A: industrially separated germ from whole grain, group B: commercially available germinated wheat germ, and group C: manually separated wheat germ from germinated grain, was studied in this work. Figure 6 shows the weight ratio of each polyamine in plant powder (polyamine concentration). Group A had the highest concentration of spermidine (220.65–337.2 μg/g), whereas Group B had the lowest concentration (19.22–24.25 μg/g). The discrepancy in polyamine content is attributable to the greater amount of starch and gluten in group B. Group C (36.45 μg/g) had less spermidine than Group A (220.65–337.2 μg/g), contrary to our expectations. After eliminating the German wheat germ from the domestic samples of group A, the mean putrescine and spermidine levels were calculated to be 109.63 and 251.66 μg/g of dried extract, respectively. When these values were compared to the values of the germinated germs (group C), it was discovered that the germination process resulted in a reduction of 34.42% putrescine and 85.52% spermidine. This can be read as 2.5 times in spermidine decrease when compared to putrescine throughout the germination phase. The discovery could aid researchers in tracking the growth of germs. The much lower polyamine content, particularly spermidine, in group C samples could be attributable to grain germination and growth, which reduced polyamine content. It is important to keep in mind that since this is the first comparison report, the assumption cannot be supported by any other sources. As a result, group A study showed that wheat germ is where polyamines are mainly stored. Our research revealed that group A, which only had wheat germ, had the highest concentration of polyamines since it lacked endosperm and bran. Inter and intra‐group differences in polyamine contents could be attributed to growing conditions, wheat variety, storage conditions, packaging, etc.

## 4 CONCLUSIONS

In conclusion, the present study provides a simple isocratic LC–MS/MS method as a practical tool for the determination of putrescine, spermidine, and spermine contents extracted from wheat germ. Three polyamines were measured successively in a quick and simple procedure with a very efficient (100%) derivatization step, avoiding the use of strong acids and common interferences found in traditional LC–MS/MS methods. Group A had the highest amounts of all three free polyamines, with putrescine having the concentration at 84.67–112.61 μg/g, followed by spermidine at 220.65–337.22 μg/g and spermine at 6.66–18.54 μg/g. A comparison of polyamine concentrations between groups A and C revealed that the germination process reduced polyamine levels of wheat germ. The highly efficient extraction protocol could be advised for nutritious formulations. In general, this study demonstrates that unprocessed wheat germ is a reliable daily source of polyamine intake. Thus, its daily administration could be suggested for individuals over 30 to promote cell proliferation, skin rejuvenation, hair and nail growth and prevent cardiovascular, chronic neurological and inflammatory diseases and malignancies. This polyamine‐rich source could also be advised for athletes, lactating women, and individuals over 30 for muscle strengthening, infant growth, and cell proliferation.

## AUTHOR CONTRIBUTIONS

**Maryam Mohajeri:** Conceptualization (equal); data curation (equal); formal analysis (equal); funding acquisition (equal); investigation (equal); methodology (equal); project administration (equal); resources (equal); software (equal); supervision (equal); validation (equal); visualization (equal); writing – original draft (equal); writing – review and editing (equal). **Seyed Abdolmajid Ayatollahi:** Conceptualization (supporting); data curation (supporting); funding acquisition (equal); investigation (equal); software (supporting); supervision (supporting); visualization (supporting); writing – original draft (supporting); writing – review and editing (supporting). **Farzad Kobarfard:** Conceptualization (equal); data curation (equal); formal analysis (equal); funding acquisition (equal); investigation (equal); methodology (equal); project administration (equal); resources (equal); software (equal); supervision (equal); validation (equal); visualization (equal); writing – original draft (equal); writing – review and editing (equal). **Mohammad Goli:** Investigation (supporting); project administration (equal); software (equal); validation (equal); visualization (equal). **Maryam Khandan:** Visualization (equal); writing – original draft (equal); writing – review and editing (equal). **Shaya Mokhtari:** Visualization (equal); writing – original draft (equal); writing – review and editing (equal). **Mahmoud Khodadoost:** Writing – review and editing (supporting).

## CONFLICT OF INTEREST STATEMENT

The authors have no conflicts of interest to declare. All co‐authors have seen and agree with the contents of manuscript and there is no financial interest to report. We certify that the submission is original work and is not under review at any other publication.

## Data Availability

The data that support the findings of this study are available from the corresponding author upon reasonable request.
